# Survey assessment on pediatricians’ attitudes on head lice management

**DOI:** 10.1186/1824-7288-39-62

**Published:** 2013-10-03

**Authors:** Claudia Fancelli, Manuela Prato, Carlotta Montagnani, Monica Pierattelli, Paolo Becherucci, Elena Chiappini, Maurizio de Martino, Luisa Galli

**Affiliations:** 1 Department of Health Sciences, Unit of Paediatrics, University of Florence, Anna Meyer Children’s University Hospital, Viale Pieraccini, 24 I-50129 Florence, Italy

**Keywords:** Head lice, Infestation, Children, Pediatrics

## Abstract

**Background:**

Pediculosis capitis is a worldwide health problem. One of the most important factor in effective head lice eradication is to ensure that infestation is adequately recognized and treated. Our survey investigated the knowledge and practice among primary care Italian pediatricians regarding to the prevention and treatment of head lice.

**Methods:**

The questionnaire was distributed to all the pediatricians registered at the Annual Congress of Practice in Pediatrics held in Florence, Italy, November 11–12, 2011. It includes 10 questions in a multiple choice format, and one answer for each question was provided. The questionnaire was conceived by pediatricians at the Infectious Disease Unit of the Department of Science for the Health of Woman and Child, University of Florence. Questions were designed according to the guidelines by the Italian Pediatric Society (SIP), and international guidelines, such as the Centers for Disease Control and Prevention (CDC), and the American Academy of Pediatrics (AAP).

**Results:**

Overall, 364/600 pediatricians (60.7% of physicians registered to the Congress) returned the questionnaire. The majority of them (232/364; 63,7%) believe that parents consult their primary care pediatrician only after the failure of other “remedies”. Mostly, they prescribe Malathion (116/364, 31,8%) as first line treatment. Two-hundred-fourty-three (66.7%) of participants consider creams, foams and gels the most effective formulations. Two-hundred-sixty-two of pediatricians interviewed (72.0%) suggest to repeat the treatment after one week, 37/364 (10.2%) after two weeks. The majority of the pediatricians interviewed reported that recurrences occur in less than 30% of cases (279/364; 76,6%). In their own opinion, most of recurrences are the consequence of a reinfestation in the community (259/264; 77%). Three-hundred-thirty-four (91.7%) of them have never prescribed oral therapy for the treatment of head lice. Finally, 289/364 (79.4%) pediatricians believe that no product is effective for prevention.

**Conclusions:**

This is the first study that investigates the clinical practice of family pediatricians about the management and treatment of head lice globally, the Italian pediatricians surveyed proved to be quite informed on the head lice management. However, even in a country where pediatric assistance is free for everybody, a considerable proportion of parents do not seek advice to their own family pediatrician. Therefore, educations of parents, other than continuous updating of pediatricians, may contribute to a better management of head lice in the community.

## Background

Pediculosis capitis is a worldwide health problem. In high resource countries, the overall prevalence of head lice infestation is 1-3%, although there is a high variability among countries. In Italy, a 2003 report exstimated an incidence of 2-3% in general population [1], while in other European countries prevalence ranges between 0.8 and 9.9% [2-7]. In the USA estimates range from 6 to 12 million cases of infestation per year [8]. Although the infestation is characterized by a low morbidity, it may cause a considerable social distress in term of discomfort to the child, absences from school and expenses incurred for treatment. Conventional topical pediculicides remain the most commonly used form of treatment globally. However, in the last 10 years, conventional therapy with topical pediculicides is increasingly associated with treatment failure and the emergence of resistance [9]. Many factors are hypothesized to cause recidives: incorrect use of pediculicides regarding to the dose and duration of the treatment, type of formulation, residual pediculicide effect, incorrect prophylaxis or intrinsic genetic resistance in lice [10-13]. However, a considerable proportion of recidives are a result of incorrect use of treatment rather than to intrinsic resistance in lice [14]. Therefore, the most important factor in effective head lice eradication is to ensure that infestation is adequately recognized and treated. Our survey investigated the knowledge and practice among primary care Italian pediatricians regarding to the prevention and treatment of head lice.

## Materials and methods

The questionnaire was distributed to all the pediatricians registered at the Annual Congress of Practice in Pediatrics held in Florence, Italy, November 11–12, 2011. This meeting is one of the most important national meeting and about 600 primary care pediatricians attend this meeting every year from all over the country.

A self-administered, anonymous questionnaire was distributed to all the pediatricians at the Congress registration desk. Overall, 600 questionnaires were distributed. The survey was administered in Italian and translated into English for publication. The English version of the questionnaire is enclosed in Additional file 1. It includes 10 questions in a multiple choice format. One answer for each question was provided. The questionnaire was conceived by pediatricians at the Infectious Disease Unit of the Department of Science for the Health of Woman and Child, University of Florence. Questions were designed according to the guidelines by the Italian Pediatric Society (SIP) [15], and international guidelines, such as the Centers for Disease Control and Prevention (CDC) [16], and the American Academy of Pediatrics (AAP) [17]. Participants were asked what kind of topical treatment they would first administer in head lice and what kind of treatment they believe to be more safe and effective in children <2 years. They were also asked to specify whether and when pediculicides treatment would be repeated, which formulation would be more effective among shampoos, powders, creams, gels, foams and sprays and the most effective attitude in preventing transmission. Final questions explored the participants’ experience and opinion towards head lice recurrence and their own attitude in case of reinfestation and infestation recurrence, including administration of oral therapy.

## Results

Overall, 364 pediatricians (60.7% of physicians registered to the Congress) returned the questionnaire. The majority of them ( 232/364; 63,7%) believe that parents ask to their own friends, their relatives or the pharmacist or reading news on Internet about the management of head lice, rather then consulting their primary care pediatrician. Moreover, we investigated the attitude towards the prescription of pediculocides in all age groups and in children younger than 2 years. Results are shown in Table 1.

**Table 1:** Lice treatment in children < 2 years of age according to the first line treatment chosen

**First line prescription treatment**		**Treatment in children <2 years**	
**Drugs**	**N (%)**	**Drugs**	**N (%)**
Permethrin	38/364 (10.4)	Permethrin	13/38 (34.2 )
		Other pirethroids and natural synergized pirethrins	3/38 (0.8)
		Malathion	1/38 (0.3)
		Natural oils	4/38 (10.5)
		Dimeticone	11/38 (28.9)
		Manual removal	4/38 (10.5)
		I don’t know	2/38 (0.5)
Other pirethroids and natural synergized pirethrins	107/364 (29.4)	Permethrin	3/107 (2.8)
		Other pirethroids and natural synergized pirethrins	51/107 (47.7)
		Malathion	0
		Natural oils	6/107 (5.6)
		Dimeticone	32/107 (29.9)
		Manual removal	12/107 (11.2)
		I don’t know	2/107 (1.9)
Malathion	116/364 (31.8)	Permethrin	1/116 (0.9)
		Other pirethroids and natural synergized pirethrins	12/116 (10.3)
		Malathion	42/116 (36.2)
		Natural oils	5/116 (4.3)
		Dimeticone	31/116 (26.7)
		Manual removal	15/116 (12.9)
		I don’t know	10/116 (8.6)
Dimeticone	76/364 (20.9)	Permethrin	0
		Other pirethroids and natural synergized pirethrins	2/76 (2.6)
		Malathion	1/76 (1.3)
		Natural oils	3/76 (3.9)
		Dimeticone	65/76 (85.5)
		Manual removal	3/76 (3.9)
		I don’t know	2/76 (2.6)
Natural oils	9/364 (2.5)	Permethrin	0
		Other pirethroids and natural synergized pirethrins	0
		Malathion	0
		Natural oils	7/9 (77.8)
		Dimeticone	0
		Manual removal	2/9 (22.2)
I don’t know	18/364 (4.9)		

Two-hundred-fourty-three (66.7%) of participants consider creams, foams and gels the most effective formulations. The remaining proportion of pediatricians believed that the most effective formulations are shampoos (72/364; 19.7%) or sprays (25/364; 6.8%) or powders (5/364; 1.4%). Two-hundred-sixty-two of pediatricians interviewed (72.0%) suggest to repeat the treatment after one week, 37/364 (10.2%) after two weeks. A minority of them (58/364; 15.9%) does not always repeat the treatment and a very low percentage (4/364; 1.1%) repeat the treatment after 4 weeks.

Moreover, the majority of the pediatricians interviewed reported that recurrences occur in less than 30% of cases (279/364; 76,6%). In their own opinion, most of recurrences are the consequence of a reinfestation in the community (259/264; 77%), rather than lice intrinsic resistance to pediculocides (22/364; 7%) or the incorrect use of these products by parents (45/364; 14%). The pediatricians attitude toward the repetition of a second full course of treatment in case of recurrence/reinfestation is shown in Table 2. Three-hundred-thirty-four (91.7%) of them have never prescribed oral therapy for the treatment of head lice. Twenty-four (6.5%) prescribed sulfamethoxazole-trimethoprim; no-one has suggested treatment with oral ivermectine. Finally, we investigated the attitude towards prevention methods. Two-hundred-eighty-nine (79.4%) pediatricians believe that no product is effective for prevention, while a minority of them suggested preventive therapy with topical pediculicides [19/364 (5.2%)], the disinfection of school and home environments [40/364 (10.9%)], and only 6/364 (1.6%) the use of hats and headgear.

**Table 2:** Percentage of recidives/reinfestations reported by pediatricians according to the first line prescription treatment suggested, the second cicle of treatment proposed, and the time range recommended for the repetition of the treatment

		**Percentage of recidives/reinfestations**		
		**Rarely or less than 10%**	**30-50%**	**I don’t know**
		**n = 237**	**n = 118**	
		**N (%)**	**N (%)**	
**First line prescription treatment**	Permethrin (38/364)	22/38 (57.9)	14/38 (36.8)	2/38 (0.1)
	Other pirethroids and natural pirethrins synergized (107/364)	71/107 (66.3)	34/107 (31.8)	2/107 (0.0)
	Malathion (116/364)	76/116 (65.5)	39/116 (33.6)	1/116 (0.0)
	Natural oils (9/364)	9/9 (100)	0	
	Dimeticone (76/364)	49/76 (64.5)	24/76 (31.6)	3/76 (0.0)
	I don’t know (5/364)			
**The second cicle of treatment suggested in case of recidive/reinfestations**	Repetition of a second complete cycle of treatment of the same class as that used previously (220/364)	143/220 (71.5)	72/220 (32.7)	5/220 (0.0)
	Repetition of a second complete cycle of treatment of different class from that used previously (131/364)	85/131 (64.95)	44/131 (33.6)	2/131(0.0)
	I don’t know (7/364)			
**Time range recommended for the repetition of the treatment**	No, not always. It depends on the product used (58/364)	44/58 (75.8)	14/58 (24.1)	0
	After 1 week (262/364)	164/262 (62.6)	93/262 (35.5)	5/262 (0.0)
	After 2 weeks (37/364)	26/37 (70.3)	10/37 (27.0)	1/37 (0.0)
	After 4 weeks (4/364)	2/4 (50.0)	0	
	I don’t know (2/364)			

## Discussion

This is the first study that investigates the clinical practice of family pediatricians about the management and treatment of head lice. Until now, several studies have focused on parental knowledges and attitudes [18-20]. Our survey agrees with all these studies showing that parents often seek advice firstly from the apothecary, or relatives, or friends, and only after a failure from a physician.

Proper education of parents is an essential component of the treatment of head lice, as well as clear treatment plans. Three options are currently described for the treatment of head lice: topical pediculocides, use of the comb and oral therapy [15-17]. The preferred topical pediculocides among these available in Italy are permethrin and other pyrethroyds, synergized pyrethrins, and malathion. In our survey, we founded that only 10.4% of the interviewed pediatricians choices permethrin as first line treatment in head lice, while about one third of pediatricians choices pirethroids and synergized pirethrins and another third of them suggests 0.5% malathion. At the time of our survey (2011), US and Italian guidelines suggested 1% permethrin as one of the treatments of choice for head lice because of its efficacy and lack of toxicity [21]. However, resistance to 1% permethrin is increasingly been reported [22-24]. Also other pirethroids (es. Phenotrin) and pirethrins synergized, which are pyrethrins combined with piperonyl butoxide, were considered as first choice by US and Italian guidelines. As regards to malathion, both the Italian and the US guidelines, published in 2010 and therefore available at the moment of our survey, suggested it as second choice, because of the odor, the flammability, and the risk of respiratory depression if ingested [15-17]. Instead, the more recent 2012 UK guidelines [25] recommend malathion as first line treatment. It’s of note that there are some differences between the European and the US formulation. First of all, the European formulation is in an aqueous basis (even with a minimum alcohol content), whereas the US formulation is more flammable, since it contains 78% isopropyl alcohol. Moreover, malathion seems to have less efficacy in Europe than in the US, probably because its continuous use in Europe during the past 30 years, whereas in US it was withdrawed in 1990 and again in 1994 and definitively reintroduced in 1999. Moreover, the formulation available only in the US (Ovide®), contains isopropanol and terpenes, which seems to have its own pediculocidal effects [26]. The results of our survey clearly show the need of frequent updating and spreading at least every 2 years national guidelines.

A further finding of our survey is that nearly 50% of pediatricians who prescribe a conventional topical pediculicide as first line therapy, administrate the same treatment in children under 24 months of age. As a matter of fact, about 40% of pediatricians choice malathion as first line treatment, and suggest this product also in children under 24 months. In general, conventional pediculicides are not recommended in children younger than 2 years [27-29]. Furthermore, malathion is not recommended in children younger than 6 years, because there are not enough studies to support its safety, and it is moreover contraindicated in children younger than 2 years [21].

It is also of note that about one fifth of pediatricians prescribe dimethicone as first line treatment and only 40.1% prescribe it in children under 24 months. In fact, in this age group, dimethicone could be an excellent therapeutic tool, since it is odourless, non-toxic and generally well tolerated by children from 6 months of age [15-17]. It acts by coating the lice and causing suffocation and has neither pediculocide nor ovocidal activity. A study in the United Kingdom reported a cure rate of nearly 70% [30], a study in Turkey reported cure rates of 92% [31], and another large randomized trial conducted in Brazil [32], in which a different formulation of dimethicone was used (92% dimethicone), reported a 97% cure rate. Maybe it is a new product, there are few studies (all from the same authors), and Italian pediatricians prescribe it less than older pediculocides. Therefore, even if 2012 UK guidelines suggest di dimethicone as first line treatment, we believe that more studies are needed to assest efficacy and safety for young children.

Furthermore, only 11% of pediatricians suggest only mechanical removal for children younger than 24 months. Wet combing is the preferred treatment for children younger than 2 years [27] and it also should be considered if parents prefer not use a pediculocide on their child. Wet combing is commonly suggested in association with topical pediculocides, in order to improve their efficacy. However, an observer-blinded study by Meinking TL, has investigated the use of 1% permethrin creme with and without adjunctive combing and it has demonstrated the failure of nit removal combing when made by non-professional caregivers [33]. Maybe, when pediatricians suggest the use of the comb, they should train parents to use the comb as appropriate [25].

Finally, very few (3%) pediatricians suggest a treatment with oils or other herbal products: their safety and efficacy are currently unknown and they are not recommended [21].

Our survey also shows that oral pediculocides (Ivermectin and Sulfamethoxazole-Trimethoprim) are, correctly, very rarely prescribed. Recently (February 2012), topical ivermectin lotion (Sklice®) was approved by the FDA. It is indicated for the treatment of head lice in children aged 6 months and older: it shows good therapeutic perspectives [34,35].

Another result of our study is that the majority of pediatrician interviewed (72%) recommend routine re-treatment for all topical pediculocides, preferably on day 7–9, as well as many experts suggest [21]. Improper timing of second application of pediculicides should be considered an important cause of treatment failure.

In literature there is a lack of data about the real incidence of treatment failure in children. The majority of pediatricians interviewed (65%) report a frequence of 10% or less of short term recidives (<2 months from treatment), while nearly one third reports a frequence rate between 30 and 50%. The majority of them believe that recidives are attributable to a re-infestation in the childhood community. More important, the ongoing presence of nits or itch is not a sign of treatment failure, since nits could be not alived. Only the finding of live lice, using a detection comb, two or three days after completing a course treatment (two applications of treatment 7 days apart) should be considered a sign of treatment failure [25]. The persistence of living head lice after the use of pediculocides may have several causes, such as: lack of adherence of the patient to the treatment protocol; inadequate dosis or duration of treatment; re-infestation (lice reacquired after treatment); and resistance of lice to pediculocides. Several studies report an increasing rate of lice resistance to topical pediculicides in the last years [13,36,37]. There are three main patterns of resistance: genetic resistance (the presence of polymorphisms in genes associated with resistance); clinical resistance (persistence of live lice after a cycle of application); parassitologic resistance (*in vivo* resistance of lice to pediculocide compounds). As Durand R describes in a recent review, permethrin-resistant phenotypes are mostly associated with a recessive *kdr* trait, while no genetical mechanism has been formally reported for malathion [26]. Now, resistance rather than a lack of compliance with treatment should be considered the main cause of a treatment failure [38]. Currently, several strategies have been proposed to overcome a possible treatment failure. One of the strategies is the application of a product for a full-course treatment and, in case of failure, the use pediculocide with a different resistance profile [17,25]. Only 37% of pediatricians interviewed used this type of therapeutic approach. Probably, the restriction of pediculocides availability only with medical prescription, the administration of these drugs at the right dose and with a correct timing may help in prevent treatment failure.

The main limit of our study is that over 40% of pediatricians attending the conference did not respond to our questionnaire and non-responders may be less updated on national guidelines as compared to responder’s pediatricians. Globally, the Italian pediatricians surveyed proved to be quite informed on head lice management. Moreover, pediatricians should advise parents that head lice infestation should be diagnosed and treated under the supervision of a physician. In fact, proper education of parents, other than continuous updating of pediatricians, may contribute to a better management of head lice in the community.

## Competing interests

The authors declare that they have no competing interests.

## Authors’ contributions

CF, MP, LG, MdM conceived of the study, performed the statistical analysis and drafted the manuscript. MP, PB, CM, EC participated in the design of the study and helped to draft the manuscript. All authors read and approved the manuscript.

## Supplementary Material

Additional file 1 Questionnaire on the management and treatment of head lice for the pediatrician. Click here for file
