# Supplementary material for: Survey assessment on pediatricians’ attitudes on head lice management
Source: Ital J Pediatr. 2013 Oct 3;39:62. doi: 10.1186/1824-7288-39-62 (PMC3853107; doi:10.1186/1824-7288-39-62)
Supplement: Additional file 1 — Questionnaire on the management and treatment of head lice for the pediatrician. [file 1824-7288-39-62-S1.doc]

Additional file 1- QUESTIONNAIRE ON THE MANAGEMENT AND TREATMENT OF HEAD LICE FOR THE PEDIATRICIAN

Dear colleague, we ask for your cooperation in answering the following questions to collect information about the management and treatment of head lice by the family pediatrician. The survey aims to describe the actual clinical practice, so please answer the following questions so sincerely and directly. The answers are anonymous. There is one answer for each question.

1. Those who believe that parents turn for advice about the management and treatment of head lice?
   1. to the family pediatrician
   2. to their friends or relatives
   3. to the pharmacist
   4. they seek informations on Internet
2. What kind of topical pediculocide do you prescribe as first line treatment?
   1. Permethrin
   2. Other pyrethroids or natural synergized pyrethrins
   3. Malathion
   4. Drugstore products (coconut extract, essential oils of anice and ylang-ylang,..)
   5. Non neurotoxic agents (dimethicone)
3. What kind of treatment do you think to be more effective and safe in children younger than 2 years?
   1. Permethrin
   2. Other pyrethroids or natural synergized pyrethrins
   3. Malathion
   4. Drugstore products (coconut extract, essential oils of anice and ylang-ylang,..)
   5. Non neurotoxic agents (dimethicone)
   6. Only mechanical removal using fine-toothed comb
4. Do you think that the treatment with topical pediculocides must always be repeated? If so, how long?
   1. No, not always. It depends on the product used
   2. Yes, after 1 week
   3. Yes, after 2 weeks
   4. Yes, after 4 weeks
5. What kind if formulation do you believe the most effective?
   1. Shampoo
   2. Powders
   3. Creams, foams, gels
   4. Spray
6. What do you think may be the most effective approach for the prevention of transmission?
   1. The use of prophylactic pediculocide products
   2. The disinfection of domestic and school environments
   3. The use of hats and caps during periods of increased transmission
   4. No product is effective for the prevention
7. In your experience, the recidive:
   1. Rarely occurs
   2. It occurs in a 10% of cases
   3. It occurs in over 30% of cases
   4. It occurs in more than 50% of cases
8. In your opinion, recidives are more frequently associated with:
   1. The incorrect application of pediculocides by family
   2. The reinfestation in the community child
   3. The intrinsic lice resistance to pediculocides
9. What is your attitude towards recidives and reinfestations?
   1. I prescribe the repetition of a second complete cycle of treatment of the same class as that used previously
   2. I prescribe the repetition of a second complete cycle of treatment of different class from that used previously
   3. I prescribe oral therapy
   4. I require a specialist dermatological assessment
10. Do you ever prescribe an oral treatment for pediculosis?
    1. No, I don’t
    2. Yes, I do. I prescribed a broad spectrum antibiotic therapy
    3. Yes, I do. I prescribed Sulfametoxazole-Trimethoprim
    4. Yes, I do, I prescribed oral Ivermectin
